# An ecological study of stillbirths in Mexico from 2000 to 2013

**DOI:** 10.2471/BLT.15.154922

**Published:** 2016-05-02

**Authors:** Teresa Murguía-Peniche, Daniel Illescas-Zárate, Gabriela Chico-Barba, Zulfiqar A Bhutta

**Affiliations:** a Faculty of Health Sciences, School of Medicine, Universidad Panamericana, Donatello 59, Colonia Insurgentes Mixcoac, Mexico City, 03920, Mexico.; b Research Division Community Interventions, Instituto Nacional de Perinatología Isidro Espinosa de los Reyes, Mexico City, Mexico.; c Center for Global Child Health, Hospital for Sick Children, Toronto, Canada.

## Abstract

**Objective:**

To examine trends in the rate of stillbirths at or after 21 weeks’ gestation in Mexico from 2000 to 2013, identify factors associated with stillbirths and estimate subnational variability in stillbirth rates and the proportion of deaths occurring intrapartum.

**Methods:**

This population-based, ecological study involved data from a national database on 263 475 stillbirths in 29 Mexican states and maternal sociodemographic factors. Subnational variability in the stillbirth rate in 2012 was investigated and stillbirths in 2013 were categorized as intrapartum or antepartum according to the fetus’ skin condition.

**Findings:**

The national stillbirth rate declined from 9.2 to 7.2 per 1000 births between 2000 and 2013 (i.e. −1.9% per year). The prevalence of stillbirths varied 3.9-fold between states. Stillbirths were associated, in particular, with: residence in Mexico City (odds ratio, OR: 1.71; 95% confidence interval, CI: 1.68–1.73) or central Mexico (OR: 1.36; 95% CI: 1.34–1.38); maternal education of 9 years or less (OR:1.10; 95% CI: 1.08–1.11) or 10 to 12 years (OR: 1.16; 95% CI: 1.14–1.18); mothers younger than 15 years (OR: 1.64; 95% CI: 1.55–1.72) or older than 34 years (OR: 1.68; 95% CI: 1.66–1.70); and male fetal sex (OR: 1.20; 95% CI: 1.19–1.21). Overall, 51% (7348/14 344) of fetal deaths occurred intrapartum.

**Conclusion:**

In Mexico, the total stillbirth rate declined between 2000 and 2013, however geographical variations were observed. Stillbirths were associated with sociodemographic factors. The proportion of intrapartum stillbirths was relatively high, suggesting that health system performance could be improved, especially at places of delivery.

## Résumé

**Objectif:**

Examiner les tendances relatives au taux de mortinatalité à 21 semaines de grossesse ou plus au Mexique de 2000 à 2013, identifier les facteurs associés à la mortinatalité et estimer la variabilité infranationale du taux de mortinatalité ainsi que la proportion de décès survenus per-partum.

**Méthodes:**

Cette étude écologique en population a utilisé des données issues d'une base de données nationale qui recensait 263 475 mortinaissances survenues dans 29 États mexicains ainsi que les facteurs sociodémographiques des mères. Nous avons analysé la variabilité infranationale du taux de mortinatalité en 2012 et classé les mortinaissances de 2013 en mortinaissances survenues per-partum ou ante-partum suivant l'état de la peau du fœtus.

**Résultats:**

Le taux de mortinatalité national est passé de 9,2 à 7,2 pour 1000 naissances entre 2000 et 2013 (soit -1,9% par an). La prévalence des mortinaissances variait de l'ordre de 1 à 3,9 selon les États. La mortinatalité était notamment associée au fait de vivre dans la ville de Mexico (rapport des cotes, RC: 1,71; intervalle de confiance (IC) de 95%: 1,68-1,73) ou dans le centre du Mexique (RC: 1,36; IC 95%: 1,34–1,38), à une durée d'instruction des mères de 9 années ou moins (RC: 1,10; IC 95%: 1,08–1,11) ou de 10 à 12 années (RC: 1,16; IC 95%: 1,14-1,18), à l’âge des mères, de moins de 15 ans (RC: 1,64; IC 95%: 1,55-1,72) ou de plus de 34 ans (RC: 1,68; IC 95%: 1,66–1,70) et au sexe masculin du fœtus (RC: 1,20; IC 95%: 1,19-1,21). Sur l'ensemble des morts fœtales, 51% (7348/14 344) étaient survenues per-partum.

**Conclusion:**

Au Mexique, le taux de mortinatalité total a baissé entre 2000 et 2013, cependant, des variations géographiques ont été observées. La mortinatalité était associée à des facteurs sociodémographiques. La proportion de mortinaissances survenues per-partum était relativement élevée, ce qui laisse penser que les performances du système de santé pourraient être améliorées, en particulier dans les lieux d'accouchement.

## Resumen

**Objetivo:**

Examinar las tendencias de las tasas de mortalidad fetal a partir de las 21 semanas de gestación en México desde 2000 hasta 2013, identificar los factores relacionados con las muertes fetales y calcular la variabilidad subnacional de las tasas de mortalidad fetal, así como el porcentaje de muertes fetales durante el parto.

**Métodos:**

Este estudio ecológico basado en la población incluyó información de una base de datos nacional de 263 475 muertes fetales de 29 estados mexicanos y factores maternos sociodemográficos. Se investigó la variabilidad subnacional de la tasa de mortalidad fetal en 2012, y en 2013 se categorizó a las muertes fetales según si el feto había fallecido durante el parto o antes del parto, de acuerdo a las características de la piel del feto.

**Resultados:**

La tasa de mortalidad fetal nacional descendió de un 9,2 a un 7,2 por cada 1 000 nacimientos entre 2000 y 2013 (es decir, -1,9% anual). La prevalencia de muertes fetales varió hasta 3,9 veces entre los estados. En concreto, las muertes fetales se relacionaron con: residencia en la Ciudad de México (razón de momios RM: 1,71; intervalo de confianza, IC, del 95%: 1,68–1,73) o centro de México (RM: 1,36; IC del 95%: 1,34–1,38); educación de la madre de 9 años o menos (RM: 1,10; IC del 95%: 1,08–1,11) o de entre 10 y 12 años (RM: 1,16; IC del 95%: 1,14–1,18); madres menores de 15 años (RM: 1,64; IC del 95%: 1,55–1,72) o mayores de 34 años (RM: 1,68; IC del 95%: 1,66–1,70); y fetos de sexo masculino (RM: 1,20; IC del 95%: 1,19–1,21). En términos generales, el 51% (7 348/14 344) de muertes fetales tuvieron lugar durante el parto.

**Conclusión:**

En México, la tasa total de mortalidad fetal descendió entre 2000 y 2013, aunque se observaron variaciones geográficas. Las muertes fetales se relacionaron con factores sociodemográficos. El porcentaje de muertes fetales durante el parto fue relativamente alto, lo que sugiere que es necesario mejorar el rendimiento del sistema sanitario, especialmente en las salas de parto.

## ملخص

**الغرض:**

دراسة النزعات السائدة في معدل وفيات الرضّع المبكرة في عمر 21 أسبوعًا من الحمل أو بعده في المكسيك في الفترة من عام 2000 إلى عام 2013، وتحديد العوامل المرتبطة بوفيات الرضّع المبكرة وتقدير التغيرات الثانوية في معدلات وفيات الرضّع المبكرة ونسبة الوفيات التي تحدث أثناء الولادة.

**الطريقة:**

تضمنت الدراسة البيئية القائمة على السكان بيانات واردة من قاعدة البيانات الوطنية بشأن 263475 حالة وفاة مبكرة للرضّع في 29 ولاية مكسيكية والعوامل الاجتماعية والديموغرافية للأمهات. وتم التحري عن التغيرات الثانوية في معدل وفيات الرضّع المبكرة في عام 2012 كما تم تصنيف وفيات الرضّع المبكرة في عام 2013 لوفيات تحدث أثناء الولادة أو في الفترة السابقة للولادة وفقًا لحالة جلد الجنين.

**النتائج:**

انخفض المعدل الوطني لوفيات الرضّع المبكرة من 9.2 إلى 7.2 لكل 1000 حالة ولادة في الفترة بين عامي 2000 و2013 (أي 1.9‏% سنويًا). وتباين معدل انتشار وفيات الرضّع المبكرة أضعاف ذلك بنسبة 3.9 ما بين الولايات. ارتبطت وفيات الرضّع المبكرة خاصة بما يلي: الإقامة في مدينة مكسيكو سيتي (نسبة الاحتمال: 1.71؛ بنسبة أرجحية مقدارها ‏95‏%: 1.68–1.73) أو منتصف المكسيك (نسبة الاحتمال: 1.36؛ ونسبة أرجحية مقدارها ‏‏95‏%: 1.34–1.38)؛ وتثقيف الأمهات لمدة 9 سنوات أو أقل (نسبة الاحتمال: 1.10؛ ونسبة أرجحية مقدارها 95‏%: 1.08–1.11) أو 10 إلى 12 سنة (نسبة الاحتمال: 1.16؛ ونسبة أرجحية مقدارها ‏‏95‏%: 1.14–1.18)؛ الأمهات الأصغر من 15 سنة (نسبة الاحتمال: 1.64؛ ونسبة أرجحية مقدارها ‏‏95‏%: 1.55–1.72) أو أكبر من 34 سنة (نسبة الاحتمال: 1.68؛ ونسبة أرجحية مقدارها ‏‏95‏%: 1.66–1.70)؛ وجنس الجنين الذكر (نسبة الاحتمال: 1.20؛ ونسبة أرجحية مقدارها ‏95‏‏%: 1.19–1.21). وبوجه عام، حدثت وفيات الأجنة بنسبة ‏51% (7348/14344) أثناء الولادة.

**الاستنتاج:**

انخفض المعدل الإجمالي لوفيات الرضّع المبكرة في المكسيك في الفترة بين عامي 2000 و2013 ولكن لوحظت اختلافات جغرافية. ارتبطت وفيات الرضّع المبكرة بالعوامل الاجتماعية والديموغرافية. كانت نسبة وفيات الرضّع المبكرة أثناء الولادة مرتفعة نسبيًا، مما يوحي بأنه من الممكن تحسين أداء النظام الصحي وخاصة في أماكن إنجاب الأطفال **. **

## 摘要

**目的:**

调查墨西哥 2000 年至 2013 年从第怀孕 21 周起出现死产率的趋势，确定与死产相关的因素并评估死产率的分区差异性以及胎儿产间死亡的比例。

**方法:**

这项基于人口的生态学研究包括来自国家数据库中 29 个墨西哥行政区 263 475 例死产数据以及产妇的社会人口因素。调查了 2012 年死产的地方性差异，并且根据胎儿的皮肤状况，将 2013 年的死产以产间或产前进行分类。

**结果:**

2000 年到 2013 年，全国每 1000 例出生死产率从 9.2 下降到了 7.2（即，每年 1.9%）。死产的普遍性在各州之间的差异是 3.9 倍。死产尤其与以下因素相关：居住在墨西哥城（比值比，OR: 1.71；95% 置信区间，CI: 1.68–1.73）或墨西哥中部 (OR: 1.36; 95% CI: 1.34–1.38)；母亲接受 9 年或少于 9 年的教育 (OR: 1.10; 95% CI: 1.08–1.11) 或 10 到 12 年的教育 (OR: 1.16; 95% CI: 1.14–1.18)；母亲小于 15 岁 (OR: 1.64; 95% CI: 1.55–1.72) 或大于34岁(OR: 1.68; 95% CI: 1.66–1.70)；男胎 (OR: 1.20; 95% CI: 1.19–1.21)。总体来说，胎儿产间死亡的比例为 51% (7348/14 344)。

**结论:**

2000 年至 2013 年，墨西哥的总死产率有所下降，然而地区性差异仍然值得注意。 死产与社会人口因素有关。产时死胎的比例相对较高，这表明卫生系统绩效有待改进，特别是在分娩的地方。

## Резюме

**Цель:**

Проанализировать тенденции изменений коэффициента мертворождаемости на 21 неделе гестации (или позднее) в Мексике за период с 2000 по 2013 год, определить факторы, обуславливающие мертворождения, и измерить вариацию коэффициентов мертворождаемости на субнациональном уровне и долю интранатальных смертей.

**Методы:**

В ходе этого популяционного экологического исследования использовались данные из национальной базы данных по 263 475 случаям мертворождения, произошедшим в 29 мексиканских штатах, и социально-демографическим факторам, определяющим состояние здоровья матерей. Была изучена вариация коэффициента мертворождаемости на субнациональном уровне в 2012 году, а мертворождения в 2013 году были отнесены к категории интранатальных или антенатальных мертворождений в зависимости от состояния кожи плода.

**Результаты:**

Национальный коэффициент мертворождаемости снизился с 9,2 до 7,2 на 1000 рождений за период между 2000 и 2013 годами (т. е. −1,9% в год). Между штатами было выявлено 3,9-кратное различие в доле мертворождений. В частности, было установлено, что вероятность мертворождения была выше, если мать проживала в городе Мехико (отношение шансов, ОШ: 1,71; 95%-й доверительный интервал, ДИ: 1,68–1,73) или в центральной части Мексики (ОШ: 1,36; 95%-й ДИ: 1,34–1,38), если продолжительность ее обучения не превышала 9 лет (ОШ: 1,10; 95%-й ДИ: 1,08–1,11) или составляла 10–12 лет (ОШ: 1,16; 95%-й ДИ: 1,14–1,18), если возраст матери был младше 15 лет (ОШ: 1,64; 95%-й ДИ: 1,55–1,72) или старше 34 лет (ОШ: 1,68; 95%-й ДИ: 1,66–1,70) и если плод был мужского пола (ОШ: 1,20; 95%-й ДИ: 1,19–1,21). В целом в 51% (7348 из 14 344) случаев гибель плода происходила интранатально.

**Вывод:**

В Мексике общий коэффициент мертворождаемости снизился за период с 2000 по 2013 год, однако это снижение различалось в зависимости от географического расположения. Мертворождения были обусловлены социально-демографическими факторами. Доля интранатальных мертворождений была относительно высокой, из чего можно заключить, что эффективность системы здравоохранения может быть увеличена, особенно в местах проведения родов.****

## Introduction

In 2015, there were an estimated 2.6 million stillbirths worldwide, where a stillbirth was the death of a fetus with a gestational age of at least 28 weeks or with a birth weight of 1000 g or more, as defined by the World Health Organization.^1^ Between 33% and 46% occurred during labour (i.e. intrapartum) and could have been prevented by simple measures.^2^^,^^3^ Between 2000 and 2015, the global stillbirth rate decreased by an estimated 2% per year from 24.7 to 18.4 per 1000 births (i.e. live or dead).^1^ In 2015, the majority of stillbirths (98%) occurred in low- and middle-income countries. The highest burden was in sub-Saharan Africa and in parts of south Asia, where the rate was 28.7 and 25.5 per 1000 births, respectively. Pakistan (43.1 per 1000 births), Nigeria (42.9 per 1000 births) and Chad (39.9 per 1000 births) had the highest rates. In contrast, six countries in Western Europe had an estimated stillbirth rate of 2 per 1000 births or less.^1^

**Fig. 1:**
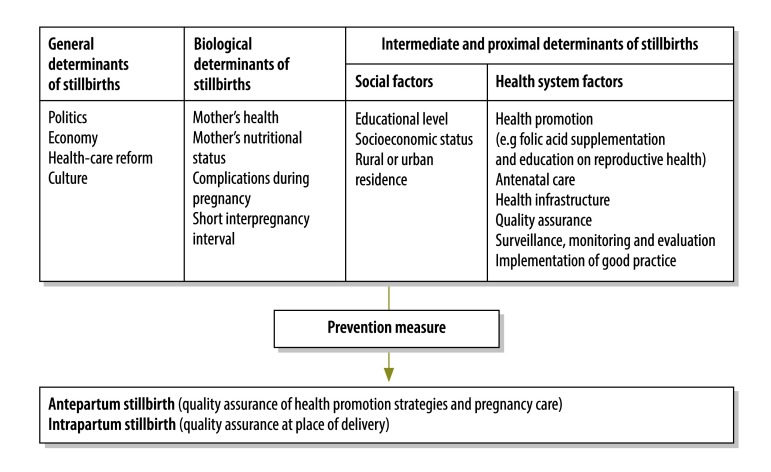
Factors influencing stillbirths

Although stillbirths are associated with a wide range of factors (Fig. 1), governments can take action to reduce stillbirths. Between 2000 and 2012, the Mexican government implemented several interventions promoting maternal and perinatal health, including Fair Start in Life,^4^ a national programme launched in 2001 that had a safe-motherhood component. In 2003, a health reform carried out under the System of Social Protection in Health established a new form of public health insurance, *Seguro Popular*. In addition, the Medical Insurance for a New Generation programme was launched in December 2006 to provide health coverage for all neonates. Further, special measures were introduced to expand coverage of antenatal care and to increase the number of institutional deliveries (i.e. the Healthy Pregnancy initiative in 2008) and to improve surveillance of maternal deaths. In 2009, the General Agreement for Collaborative Interinstitutional Care of Obstetric Emergencies was established.^5^^,^^6^ Subsequently, maternal mortality declined to 49.0 per 100 000 live births in 2014 from 90.4 per 100 000 in 1990.^7^^,^^8^

However, despite the importance of these interventions for public health, their impact on fetal outcomes has yet to be analysed and, in particular, few data on stillbirths have been reported for Mexico. The failure to count stillbirths makes it impossible to investigate their effect on families, on parental mental health, on economic and psychosocial development or on the health system.^9^ In fact, counting and auditing fetal deaths are crucial for understanding perinatal outcomes and for implementing strategies to improve them. Moreover, the perinatal mortality rate is an indicator of quality of care and is essential for making international comparisons.^10^

The objectives of this study were: (i) to analyse trends in stillbirth rates in Mexico from 2000 to 2013; (ii) to identify sociodemographic factors associated with the stillbirth rate during this period; (iii) to investigate subnational variations in the rate; and (iv) to determine the proportion of stillbirths that occurred intrapartum in 2013. We hypothesized that perinatal outcomes have improved in Mexico over recent years because the country has implemented several programmes with that intent.

## Methods

Our study was a population-based ecological study covering 2000 to 2013. We used aggregated data from the Mexican National Institute of Statistics and Geography, which records information on vital statistics every year and census data in selected years. In Mexico, about 95% of births occur in hospitals, which register live births and issue death certificates.^11^ Consequently, our data represent population estimates. The information we obtained included fetal birth weight, fetal gestational age from the last menstrual period and selected maternal sociodemographic factors. We defined a stillbirth as a fetal death that occurred at a gestational age of 21 weeks or more: stillbirths were further classified as early (i.e. occurring between 21 and 27 weeks’ gestation) or late (i.e. occurring at 28 weeks’ gestation or later). We used information for 2013 on the condition of the stillborn fetus’s skin at birth to determine when death occurred: fresh skin was taken to indicate an intrapartum death, whereas macerated skin indicated an antepartum death.^12^ The percentage of intrapartum stillbirths was stratified by gestational age. In addition, information about the health system was obtained from the Directorate General of Health Information, which maintains public databases.^13^

Stillbirth rates were expressed as the number of fetal deaths per 1000 births (i.e. live or dead). We compared the stillbirth rate in each state for each year between 2000 and 2013 with the corresponding neonatal mortality rate, which was defined as the number of neonatal deaths per 1000 live births. As suggested by others,^2^ we excluded any state in which the ratio of the stillbirth rate to the neonatal mortality rate in any year was less than 0.25 or more than 4.0 because such a rate was implausible, given that the expected ratio should be close to 1, and probably indicated poor reporting. Data on economic indicators, such as the gross domestic product (GDP) per capita, the Gini index and the human development index, were obtained from the National Institute of Statistics and Geography for the year 2012 – the most recent year for which official data on all economic indicators were available. Any state with a GDP per capita more than four standard deviations above the mean for all states was considered an outlier and was excluded from the analysis.

We obtained sociodemographic data on the entire Mexican population from the 2000, 2005 and 2010 censuses. The censuses included national information on: (i) the percentage of all pregnancies that occurred in teenage women (i.e. women aged 19 years or younger); (ii) the percentage of women who lived in an urban or rural area; and (iii) the percentage of women of reproductive age who had had either less than 6 years, 7 to 9 years, 10 to 12 years or more than 12 years of education. In addition, we obtained national health system information on: (i) the number of hospitals per 1 000 000 population (2005 and 2010 censuses only); (ii) the proportion of women who had a live birth and had had more than four antenatal visits (2010 census only); and (iii) the proportion of deliveries assisted by a skilled birth attendant (2010 census only). In our analysis of stillbirths, we considered the influence of the sex of the fetus, urban or rural residence, maternal age (i.e. younger than 15 years, 15 to 19 years, 20 to 34 years or older than 34 years) and the mother’s educational level (i.e. 9 years or less, 10 to 12 years, or more than 12 years of education). The place of delivery was categorized as a private institution, a public institution or other (i.e. outside an institution). For the study, Mexican states were divided into four regions: northern, central, Mexico City and southern, as described previously.^14^

### Statistical analysis

We analysed the trend in early and late stillbirth rates between 2000 and 2013 and, for comparison, the trend in the neonatal mortality rate. The compound annual percentage rate change was calculated as:

$$\left[{\left(a/b\right)}^{\left(1/c\right)}-1\right]\;\times \;100\;$$ (1)

where *a* defines the most recent stillbirth rate, *b* defines the earliest stillbirth rate and *c* defines the number of years. 

A sequential analysis technique involving a two-sided cumulative sum control chart was used to detect changes in the national stillbirth rate between 2000 and 2013 that were greater than one standard deviation from the national mean stillbirth rate for that period overall, in either a positive or negative direction. The changes were plotted over time in a diagram with two converging horizontal boundaries. If the fetal death rate dropped below the lower boundary, the rate was higher than expected; if it rose above the upper boundary, it was lower than expected.

We were unable to calculate 95% confidence intervals (CIs) for the national prevalence of sociodemographic factors and health system indicators for 2000, 2005 and 2010 because of limitations in the data sources. However, stillbirth rates were calculated for the period 2000 to 2013 for a range of sociodemographic subgroups and for different places of delivery. By using crude odds ratios (ORs) and 95% CIs derived from these rates, we were able to identify factors associated with fetal deaths. Odds ratios were not calculated for any variable for which 20% or more possible data values were missing. We were unable to perform a multivariable analysis because our sources provided aggregated data rather than data on individuals. Moreover, the National Institute of Statistics and Geography’s database did not include information on the number of antenatal care visits received by a mother for each live birth before 2008. We compared stillbirth rates in different Mexican states in 2012 and derived Spearman correlation coefficients for the relationship between the stillbirth rate and the state’s GDP per capita, Gini index and human development index. OpenEpi version 3.03 was used to calculate ORs, rates and prevalences and other analyses were performed using SAS v. 9.4 (SAS Institute Inc., Cary, United States of America). *P*-values less than 0.05 were considered significant.

## Results

**Fig. 2:**
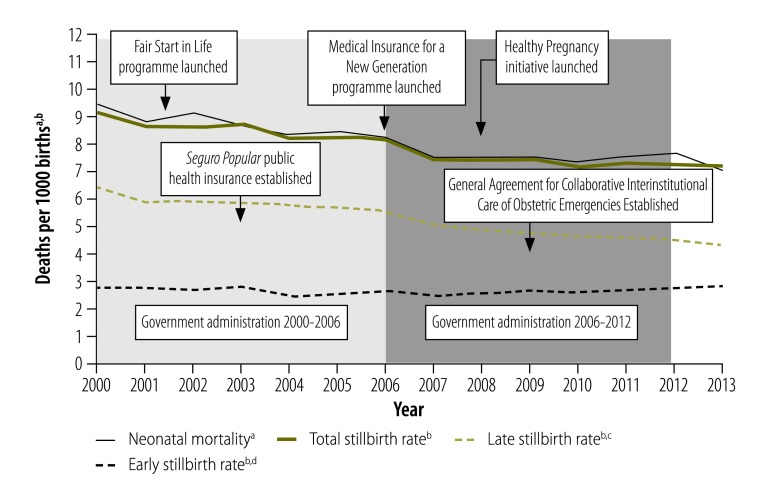
Stillbirth and neonatal mortality rates, Mexico, 2000–2013

^a^ Neonatal mortality is reported in deaths per 1000 live births.

^b^ Stillbirth rates are reported in deaths per 1000 births (i.e. live or dead).

^c^ A late stillbirth was one occurring at 28 weeks’ gestation or later.

^d^ An early stillbirth was one occurring between 21 and 27 weeks’ gestation.

**Fig. 3:**
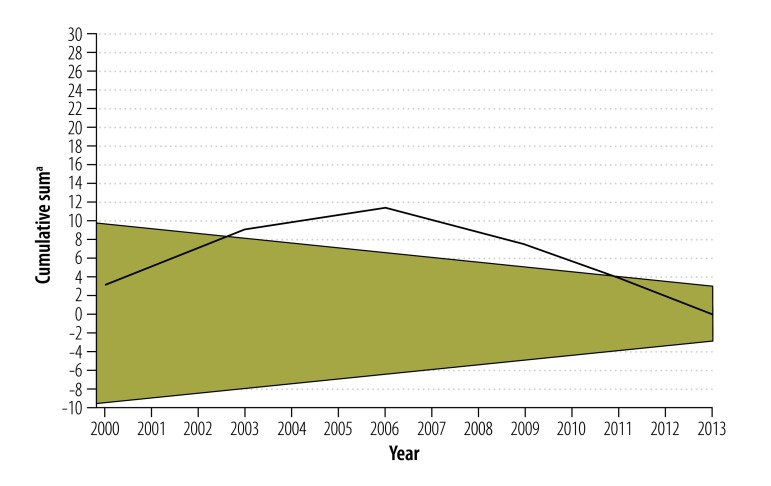
Cumulative sum control chart for the stillbirth rate, Mexico, 2000–2013

^a^ The *y*-axis scale indicates the cumulative sum of the standardized deviation of the mean in each year from the national mean stillbirth rate for 2000 to 2013 overall. The analysis was set to identify values more than one standard deviation from the mean stillbirth rate for the whole period with an α of 0.10. In particular, the stillbirth rate was lower than expected in years where the line lies above the upper boundary of the shaded area.

**Fig. 4:**
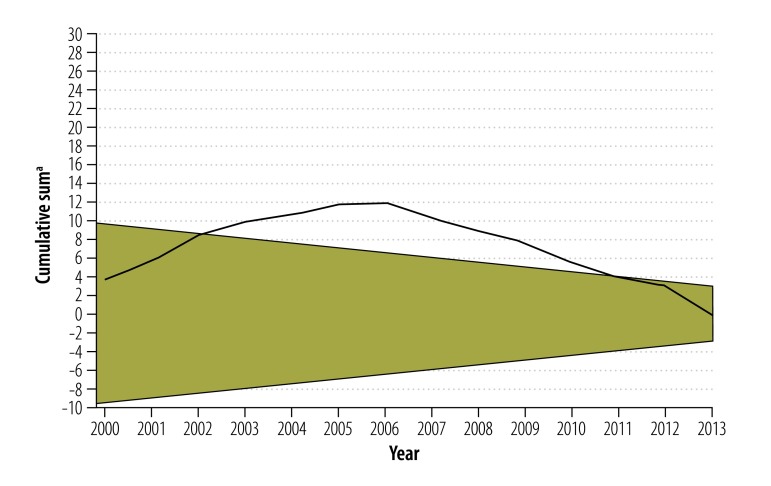
Cumulative sum control chart for neonatal mortality, Mexico, 2000–2013

^a^ The *y*-axis scale indicates the cumulative sum of the standardized deviation of the mean in each year from the national mean neonatal mortality rate for 2000 to 2013 overall. The analysis was set to identify values more than one standard deviation from the mean neonatal mortality rate for the whole period with an α of 0.10. In particular, neonatal mortality was lower than expected in years where the line lies above the upper boundary of the shaded area.

Our analysis included data from 29 of the 32 Mexican states: Coahuila, Guerrero and Sinaloa were excluded because some stillbirth-to-neonatal mortality ratios lay outside the range 0.25 to 4.0. In 2013, these three states accounted for 8.8% (218 087/2 478 889) of all births nationally and for 2.6% (431/16 440) of all stillbirths. A total of 33 163 577 births and 263 475 stillbirths occurred between 2000 and 2013 in the other 29 states (average stillbirth rate: 7.9 per 1000 births). Fig. 2 depicts the national trend in stillbirth and neonatal mortality rates over that period and indicates the years in which various national strategies to improve perinatal outcomes were implemented. The compound annual decline in these two rates was −1.9% and −2.2%, respectively. The late fetal death rate decreased by an average of −3.0% per year, whereas the early fetal death rate increased slightly by 0.2% per year. According to the cumulative sum control chart analysis (Fig. 3 and Fig. 4; both available at: http://www.who.int/bulletin/volumes/94/5/15-154922), the trends in fetal and neonatal mortality rates declined more than expected between 2003 and 2010.

Table 1 lists selected sociodemographic characteristics for the whole Mexican population in 2000, 2005 and 2010 and several health system variables. The proportion of women with more than 12 years of education and the number of public hospitals per 1 000 000 population both increased substantially between 2000 and 2010. The proportion of pregnancies occurring in teenagers and in women aged over 34 years increased. Table 2 shows how the number and rate of stillbirths varied according to the sex of the fetus, place of delivery and various demographic characteristics for the whole study period. Table 3 lists the factors associated with stillbirths. Odds ratios were not calculated for the place of delivery or the area of residence because a large proportion of data was missing.

**Table 1:** Sociodemographic and health system characteristics, Mexico, 2000, 2005 and 2010

Sociodemographic and health system characteristic^a^	Year			Change from 2000 to 2010, (%)
	2000	2005	2010	
**Population^b^**				
National population, no.	97 483 412	103 263 388	112 336 538	15.24
Urban residents, %	74.60	76.50	76.80	2.95
Rural residents, %	25.40	23.50	23.20	−8.66
Women aged > 15 years, no.	32 798 814	36 019 758	40 767 055	24.30
Total births^c^ no.	2 798 339	2 567 906	2 643 908	–5.51
Live births, no.	2 766 375	2 539 533	2 597 767	–6.27
**Proportion of women aged > 15 years educated for^b,d^**				
≤ 6 years, %	49.80	42.40	37.30	−25.10
7–9 years, %	22.90	24.80	26.50	15.72
10–12 years, %	17.10	18.60	19.30	12.87
> 12 years, %	9.40	12.40	15.90	69.15
**Mean duration of women’s education, years**	7.20	7.90	8.50	18.06
**Proportion of women who became pregnant aged^b^**				
< 15 years, %	0.44	0.38	0.44	0
15–19 years, %	15.49	16.06	17.55	13.29
20–34 years, %	67.99	68.73	67.76	−0.34
> 34 years, %	9.46	9.52	9.87	4.33
**Health system infrastructure**^e^				
All hospitals, no.	ND	3803	4388	15.38^f^
All hospitals per 1 000 000 population	ND	36.83	39.06	6.06^f^
Public hospitals, no.	ND	630	1244	97.46^f^
Public hospitals per 1 000 000 population	ND	6.10	11.07	81.51^f^
Private hospitals, no.	ND	3173	3144	−0.91^f^
Private hospitals per 1 000 000 population	ND	30.73	27.99	−8.92^f^
**Proportion of pregnant women who had^e^**				
< 4 antenatal care visits, %	ND	ND	10.73	NA
≥ 4 antenatal care visits, %	ND	ND	89.27	NA
**Proportion of women who gave birth assisted by a skilled birth attendant^g^_,_ %**	ND	ND	97.78	NA

NA: not applicable; ND: not determined.

^a^ Data are for the entire Mexican population.

^b^ Data from the Mexican National Institute of Statistics and Geography.

^c ^Total births includes live and dead births, national and foreign-born babies (foreign born: n = 8120, 8190 and 30 297 for years 2000, 2005 and 2010 respectively).

^d^ The sum of the percentages of women educated for different times does not total 100% because of missing values.

^e^Data from the Directorate General of Health Information.^13^

^f^ Percentage change from 2005 to 2010.

^g^ In the data source, physicians, nurses and other health-care workers were included as skilled birth attendants but midwives were not.

**Table 2:** Stillbirths, by demographic characteristic, Mexico, 2000–2013

Demographic characteristic^a^	Stillbirths, no.^b^	Births, no.^b^	Stillbirths per 1000 births (95% CI)
**National total**	263 475	33 163 577	7.94 (7.91–7.97)
**Sex of fetus**			
Female	117 938	16 533 893	7.13 (7.09–7.17)
Male	141 267	16 589 422	8.51 (8.47–8.56)
Unknown, %	1.62	0.12	NA
**Region of country**			
Northern	40 036	6 211 015	6.45 (6.38–6.50)
Central	116 169	13 279 387	8.75 (8.69–8.78)
Mexico City	30 949	2 823 854	10.96 (10.84–11.08)
Southern	76 321	10 849 321	7.03 (6.98–7.09)
**Maternal age at delivery, years**			
< 15	1 476	125 401	11.77 (11.17–12.37)
15–19	40 628	5 473 878	7.42 (7.35–7.49)
20–34	163 724	22 661 684	7.22 (7.19–7.26)
> 34	38 502	3 192 736	12.06 (11.94–12.18)
Unknown, %	7.27	5.16	NA
**Maternal education, years**			
≤ 9	183 076	22 073 363	8.29 (8.25–8.33)
10–12	43 001	4 912 778	8.75 (8.67–8.83)
> 12	21 196	2 802 434	7.56 (7.46–7.66)
Unknown, %	6.15	10.18	NA
**Area of residence^c^**			
Rural	53 441	3 699 066	14.45 (14.33–14.57)
Urban	193 730	22 851 198	8.48 (8.44–8.51)
Unknown, %	6.19	19.94	NA
**Place of delivery^c^**			
Public institution	129 384	21 306 009	6.07 (6.04–6.10)
Private institution	27 058	5 303 970	5.10 (5.04–5.16)
Outside an institution	11 102	4 034 635	2.75 (2.70–2.80)
Unknown, %	36.41	7.60	NA

CI: confidence interval; NA: not applicable.

^a^ Data are for 29 of the 32 Mexican states – Coahuila, Guerrero and Sinaloa were excluded because the stillbirth-to-neonatal mortality ratio was outside the range 0.25 to 4.0.

^b^ All values are absolute numbers unless otherwise stated.

^c^ Data for 2004 to 2013 only.

**Table 3:** Factors associated with stillbirths, Mexico, 2000–2013

Factor	Risk of stillbirth, crude OR (95% CI)
**Sex of fetus**	
Female	Reference
Male	1.20 (1.19–1.21)
**Region of country**	
Northern	Reference
Central	1.36 (1.34–1.38)
Mexico City	1.71 (1.68–1.73)
Southern	1.09 (1.08–1.10)
**Maternal age at delivery, years**	
< 15	1.64 (1.55–1.72)
15–19	1.03 (1.02–1.04)
20–34	Reference
> 34	1.68 (1.66–1.70)
**Maternal education, years**	
≤ 9	1.10 (1.08–1.11)
10–12	1.16 (1.14–1.18)
> 12	Reference

CI: confidence interval; OR: odds ratio.

**Fig. 5:**
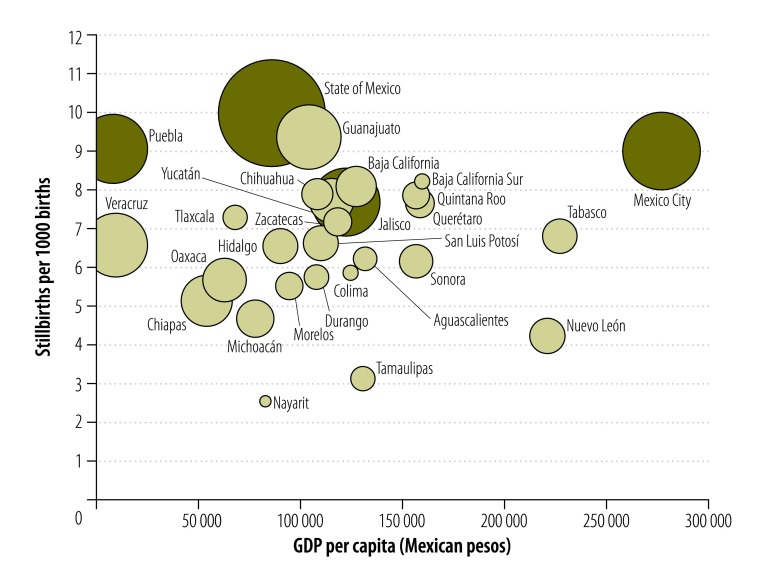
Stillbirths in 28 states, by gross domestic product per capita, Mexico, 2012

Fig. 5 depicts the stillbirth rate and the number of stillbirths in 2012 in 28 of the 29 Mexican states included in the analysis and illustrates their relationship with GDP per capita. The state of Campeche was excluded because its GDP per capita was more than four standard deviations above the mean. Four of the 28 states – the State of Mexico, Mexico City, Puebla and Jalisco – accounted for 44% (7098/16 210) of all stillbirths. These four states were home to about one third of the national population. There was no clear correlation between a state’s GDP per capita and its stillbirth rate. Moreover, there was no apparent correlation between the stillbirth rate and either the Gini index or the human development index across the states: the correlation coefficient was −0.180 (*P* = 0.36) and 0.122 (*P* = 0.53) for the two indices, respectively. However, there was a 3.9-fold difference in the stillbirth rate between the State of Mexico, which had the highest rate, and Nayarit, which had the lowest.

GDP: gross domestic product.

Notes: The diameter of the circles is proportional to the number of stillbirths in the state. The dark green circles represent the four states with the highest stillbirth rates, which together accounted for 44% of all stillbirths in the country. The states of Coahuila, Guerrero and Sinaloa were excluded because the stillbirth-to-neonatal mortality ratio was outside the range 0.25 to 4.0 and Campeche was excluded because its GDP per capita was more than four standard deviations above the mean for all states.

**Fig. 6:**
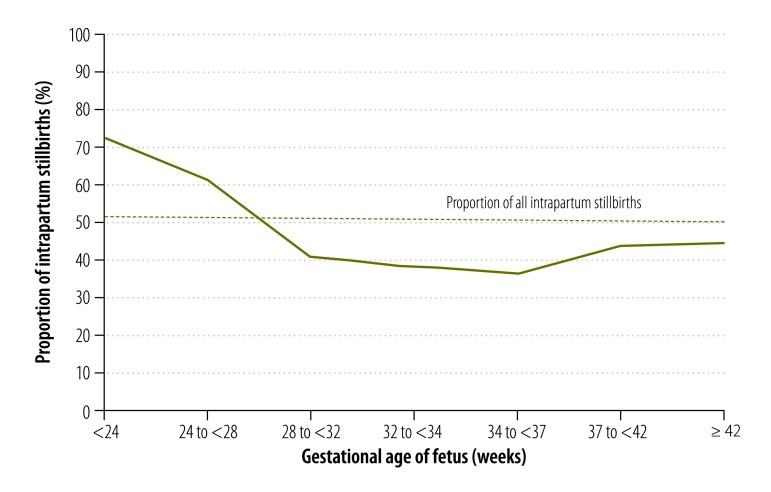
Proportion of stillbirths occurring intrapartum, by gestational age, Mexico, 2013

We analysed the time of fetal death for 16 009 stillbirths reported in 2013 – 1665 were excluded because their skin condition was not reported. Among stillbirths whose skin condition was known, the mean proportion of deaths that occurred intrapartum was 51% (7348/14 344): the proportion varied from 34% (28/82) to 57% (886/1542) across the states. In addition, the proportion of deaths that occurred intrapartum varied with the gestational age of the fetus (Fig. 6). The proportion was 72% (2098/2907) for stillbirths that occurred earlier than 24 weeks’ gestation and was roughly 40% for those that occurred at 28 weeks’ gestation or later.

## Discussion

In Mexico, the decreases in stillbirth and neonatal mortality rates observed between 2000 and 2013 were similar, which was expected because the factors affecting these two outcomes are closely linked. However, the neonatal mortality rate increased slightly between 2009 and 2012, whereas the total stillbirth rate declined (Fig. 2). Moreover, whereas the early (i.e. 21 to 27 weeks’ gestation) stillbirth rate failed to improve, the late (i.e. 28 weeks’ gestation or more) rate declined continuously. These observations might indicate that obstetric care improved or that at-risk fetuses were detected and delivered early, thereby increasing the burden of care on neonatologists who would have had to treat smaller and sicker babies. These findings deserve further research.

Overall, the stillbirth rate declined by 24.3% between 2000 and 2013 from 9.2 to 7.2 per 1000 births, thereby saving an estimated 41 630 fetuses over this period. Worldwide, the average estimated stillbirth rate after 28 weeks’ gestation in 2015 was 18.4 per 1000 births, which corresponded to a decline of 25.5% from 2000.^1^ In China, the decline between 1995 and 2009 was a remarkable 47.5%, whereas the decline in countries in sub-Saharan Africa and Oceania during the same period was smaller, at around 8%.^2^ However, countries define stillbirths differently. For example, previous global estimates were based on stillbirths in the third trimester whereas we included all fetal deaths after 21 weeks’ gestation. Considering only third-trimester fetuses, the stillbirth rate in Mexico decreased from 6.4 to 4.3 per 1000 births between 2000 and 2013 (i.e. compound annual rate: −3.0%). This decline is greater than in Latin America as a whole, where the stillbirth rate decreased from 11.3 to 8.2 per 1000 births between 2000 and 2015 (i.e. compound annual rate: −2.1%).^1^^,^^2^

In Mexico, several factors affecting the stillbirth rate improved during the study period. In our view, changes in general determinants of fetal health, such as government policy and health-care reform (Fig. 1), were important for improving perinatal outcomes – in particular, the cumulative sum control chart analysis showed that fetal outcomes were better than expected between 2003 and 2010. Government strategies aimed at expanding pregnancy and emergency obstetric care appeared to decrease the stillbirth rate, though a plateau was reached at the end of the government administration from 2006 to 2012. The two main strategies were: (i) health reform legislation in 2003 that created the System of Social Protection in Health; and (ii) the launch of the Medical Insurance for a New Generation programme in 2006.^5^ As a result of all strategies: (i) 51.8 million of the more than 60 million Mexicans who had no institutional health protection in 2002 were enrolled in *Seguro Popular* by 2011; (ii) the physician-to-population ratio increased 54% between 2004 and 2010 such that there were 46.2 health-care providers per 10 000 people in 2012; (iii) the proportion of mothers using public health facilities (ministry of health) to give birth increased from 32% to 48% between 2000 and 2012; (iv) the proportion of pregnant women who received antenatal care increased from 67.3% to 89.2% between 2006 and 2010; and (v) the proportion of deliveries aided by a skilled birth attendant reached 97.8% in 2010.^5^^,^^8^^,^^13^ In addition, the educational level of women of reproductive age increased during the study period and the health infrastructure improved. Together these interventions could explain some of the improvement in fetal outcomes observed during the two government administrations between 2000 and 2012, which illustrates that modifying the social determinants of health and pregnancy care is important. Nevertheless, attention must be paid to the quality of care if fetal well-being is to be further enhanced.

Fetal outcomes were better in northern parts of the country and in urban areas. This could be due to environmental, genetic or sociodemographic factors or to a failure of the health-care system in some areas. In addition, the stillbirth rate was higher in women who became pregnant very early or very late in their reproductive life and in those with a lower level of education. Multisectoral efforts are needed to empower and educate women and to promote pregnancies at an optimal maternal age. The odds of dying in utero were higher for a male than a female fetus, as previously reported.^15^ We observed up to a 3.9-fold difference in the stillbirth rate among states in 2012. This large variation should be addressed by the health system and political leaders. We failed to find a correlation between the stillbirth rate and the GDP per capita, Gini index or human development index across Mexican states. This was unexpected but may be related to the good health-care coverage achieved in Mexico by government policies. Alternatively, deaths may have been underreported in regions with fewer resources.^16^ The four states with the highest observed burden of stillbirths deserve further study.

In Mexico, 51% of all stillbirths occurred intrapartum, as did around 40% of stillbirths reported at 28 weeks’ gestation or later. The latter figure is within the global estimate of 33 to 46% for third-trimester stillbirths.^2^^,^^3^ These proportions are very high and action should be taken to reduce stillbirth rates both in Mexico and worldwide. Since most deliveries in Mexico take place in institutions,^11^ efforts could be made to improve emergency obstetric care and monitoring practices during labour. In fact, according to a Delphi analysis conducted by experts, providing basic emergency obstetric care could reduce intrapartum deaths by 40% compared with not providing such care and providing comprehensive emergency obstetric care could reduce deaths by 85%.^17^ Recently, two global initiatives have targeted the reduction of stillbirths – Stillbirths: the vision for 2020 and the Every Newborn action plan.^18^^,^^19^ The first calls for countries with a third-trimester stillbirth rate under 5 per 1000 births to eliminate all preventable stillbirths and close equity gaps by 2020. All other countries should reduce their stillbirth rate by at least 50% by 2020 from a 2009 global average of 18.9 per 1000 births.^2^^,^^18^ The second initiative adopted a national stillbirth target rate of 10 per 1000 births or lower by 2035, advocated addressing inequalities such as subnational variations in the stillbirth rate and encouraged the use of strategies to avoid preventable deaths.^19^

One strength of our analysis is the use of data from a large, representative, national database that covered two government administrations, during which several health programmes and initiatives were implemented. Furthermore, by analysing the skin condition of fetuses as a proxy for the time of death, we were able to identify the best intervention strategies. Limitations included the underreporting of stillbirths in three states and the absence of information about the fetus’s skin condition on around 10% of death certificates. Moreover, because several public health interventions overlapped during the study period, it was difficult to assess their individual effects. The missing data on some sociodemographic characteristics influencing stillbirths might have affected our findings. Also, since it was not possible to match live births and death certificates in the national health information system, an adjusted analysis could not be performed.

In conclusion, implementation of strategies to improve social conditions and pregnancy care has most likely decreased the stillbirth rate in Mexico. However, to further prevent antenatal deaths, more interventions are needed – such as improvement in education, good prenatal and preconception interventions and improvement of the quality of care at places of delivery.
